# Optimizing clinical outcomes in polypharmacy through poly-de-prescribing: a longitudinal study

**DOI:** 10.3389/fmed.2024.1365751

**Published:** 2024-04-30

**Authors:** Doron Garfinkel, Yuval Levy

**Affiliations:** ^1^Center for Appropriate Medication Use, Sheba Medical Center, Ramat Gan, Israel; ^2^Sheba Medical Center, Ramat Gan, Israel

**Keywords:** poly-de-prescribing, polypharmacy, inappropriate medication use, geriatric palliative approach, multi-morbidity, dementia, frailty

## Abstract

**Objectives:**

To evaluate polypharmacy in older people to determine whether the number of medications de-prescribed correlates with the extent of improvement in quality of life (QoL) and clinical outcomes.

**Design:**

A prospective longitudinal cohort study of polypharmacy in people living in a community in Israel.

**Setting:**

Participants aged 65 years or older who took at least six prescription drugs followed up for at least 3 years (range 3–10 years) after poly-de-prescription (PDP) recommendations.

**Interventions:**

PDP recommended at first home visit using the Garfinkel algorithm. Annual follow-up and end-of-study questionnaires used to assess clinical outcomes, QoL, and satisfaction from de-prescribing. All medications taken, complications, hospitalizations, and mortality recorded. In total, 307 participants met the inclusion criteria; 25 incomplete end-of-study questionnaires meant 282 participants for subjective analysis. Participants divided into two subgroups: (i) those who discontinued more than 50% of the drugs (PDP group) or (ii) those who discontinued less than 50% of the drugs (non-responders, NR).

**Main outcome measures:**

Objective: 3-year survival rate and hospitalizations. Subjective: general satisfaction from de-prescribing; change in functional, mental, and cognitive status; improved sleep quality, appetite, and continence; and decreased pain.

**Results:**

Mean age: 83 years (range 65–99 years). Mean number of drugs at baseline visit: 9.8 (range 6–20); 6.7 ± 2.0 de-prescribed in the PDP group (*n* = 146) and 2.2 ± 2.1 in the NR group (*n* = 161) (*p* < 0.001).

No statistical difference between the groups in the 3-year survival rate and hospitalizations, but a significant improvement in functional and cognitive status and, in general, satisfaction from the intervention in the PDP group compared to the NR group. Improvement usually evident within the first 3 months and persists for several years.

**Conclusion:**

Poly-de-prescribing in the older population has beneficial effects on several clinical outcomes with no detrimental effect on the rate of hospitalization and survival. The extent of improvement correlates with the extent of de-prescribing. Applying the Garfinkel algorithm globally may improve QoL in millions of patients, a clinical and economic win–win situation.

## Introduction

Advanced successful healthcare systems, despite their many advantages, also brought about a “tidal wave” of inappropriate medication use and polypharmacy (IMUP) as a result of a phenomenal rise in the number of specialists and medications, as well as over-diagnosis. IUMP may be problematic for vulnerable populations, in particular for the VOCODFLEX (a term previously coined by us) group that represents Very Old people, with COmorbidity, Dementia, Frailty/disability, and with limited Life EXpectancy (1–3). Quite clearly, IMUP has become a worldwide problem. Unlike pandemics, for which immunization and/or treatment is rapidly found within several years, no consensus exists regarding the best way to address the problem of IMUP and we seem to be losing the war against this insidious, century-old iatrogenic condition (1–4).

The reason for this medical failure is probably multifactorial and involves fundamental mistakes in our traditional research and clinical perceptions, conflicts of interest, and psychological inhibitions in both health professionals and the general public. A series of efforts and monetary resources have been expended for several decades to find ways to suppress IMUP (5–10). Unfortunately, these efforts led to only minor improvements in clinical outcomes, with no large absolute reduction in drugs, if any (2, 4, 11). Lack of evidence supporting the benefits of de-prescribing may explain why many physicians, although being aware of the harmful consequences of IMUP, are reluctant to routinely de-prescribe (12).

Our hypothesis is that the clinical harm resulting from IMUP outweighs the sum total of all the beneficial effects of the specific drugs and combinations of drugs de-prescribed.

The main determinant of IMUP is the absolute number of drugs (13–17). The present study was designed to evaluate the effect of long-term de-prescribing on the quality of life (QoL), clinical outcomes, survival, and rate of hospitalizations in older people. To our knowledge, this is also the first longitudinal study attempting to establish that, regardless of the types of drugs discontinued, the extent of de-prescribing itself correlates with the extent of benefit in clinical outcomes and QoL.

## Methods

This longitudinal cohort study included patients living in a community and over 65 years of age, who were taking at least six prescription drugs that did not include vitamins, minerals, food additives, topical preparations, and over-the-counter medications. Patients were referred to the consultant geriatrician (DG) for a comprehensive geriatric assessment (CGA) or specifically for de-prescribing. Exclusion criteria were life expectancy shorter than 6 months and the inability of the patient or their family to adhere to an orderly, long-term follow-up. Patients were enrolled in the study beginning in 2009 and all were followed up for at least 3 years until 2019 (3–10 years follow-up).

### Baseline visit

During the first visit, the geriatrician resorted to a detailed data collection and performed CGA, including an evaluation of all the prescription and non-prescription medications. All patients were subjected to a full physical examination and an up-to-date laboratory evaluation. Functional status was determined using a 5-point scale, modified from the traditional Fried’s phenotype model (18):1 = independent, 2 = frail 3 = mild disability [needs help in 1–2 activities of daily living (ADLs)]; 4 = disability (needs help with at least 3 ADLs); 5 = severe disability/bedridden. Cognitive status was assessed using the Mini Mental State Examination (MMSE) test. Depression was assessed using the Geriatric Depression Scale (GDS) short form except for patients with severe dementia.

### Poly-de-prescribing process

**Figure 1:**
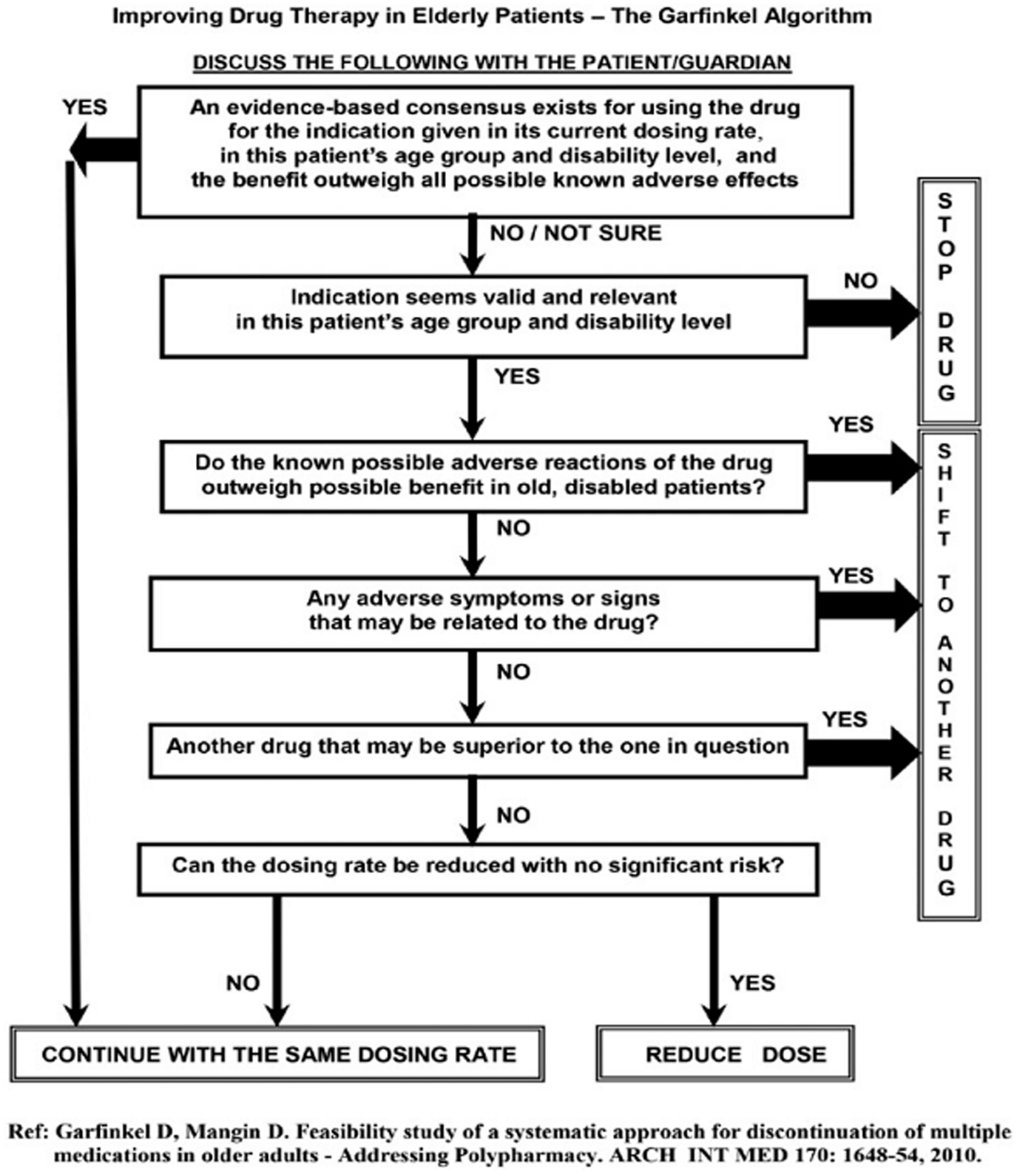
Improving drug therapy in older people: the Garfinkel algorithm.

De-prescribing of medications was done using the Garfinkel method; it postulates that in older people, the appropriateness of continuing each drug on a patient’s prescription list should be thoughtfully considered. This is done using the Garfinkel algorithm de-prescribing tool (Figure 1) (19, 20). The method combines evidence-based medicine research data (when exists) with particular characteristics of the patient/family (e.g., values, beliefs, and functional and cognitive status), placing their preferences as the highest priority. Improving QoL as perceived by the patient/family takes precedence over achieving chronic disease care targets (e.g., blood pressure [BP] level, serum glucose, and lipid concentrations). Collaboration with the family/patient is therefore central to the Garfinkel method, which requires devoting time to addressing their concerns and providing explanations. Taking into consideration the known literature for each drug and risks of polypharmacy, we advise the patient/family on poly-de-prescribing (PDP) recommendations and receive their consent to stop as many non-life-saving drugs as possible. These may include preventative medications (e.g., antihypertensive medications [AHT], cholesterol-lowering drugs, aspirin, anticoagulants), as well as drugs for relieving symptoms such as sleeping pills, drugs for dyspepsia, or vertigo. Drugs from different groups are discontinued simultaneously while drugs prescribed for the same indication (e.g., AHT) are stopped one drug at a time with a detailed plan. Detailed verbal and written recommendations are provided to the patient/family, along with supporting references to the family doctor/general practitioner (GP). In this study, all participants were given individual recommendations for PDP from the same geriatrician.

### Follow-up

All patients/families were contacted by phone at least once a year and their comments recorded regarding their health status and any change in symptoms and signs. Furthermore, every drug that was discontinued was also followed up for undesirable adverse effects (AE). For example, when discontinuing proton pump inhibitors (PPIs) or H2 blockers, participants were followed for gastrointestinal bleeding or dyspepsia; when medications for Parkinson’s disease were gradually de-prescribed, patients were evaluated for deterioration in extrapyramidal symptoms/signs and/or functional decline.

In all the participants, an end-of study questionnaire (Appendix 1) was administered between March 2017 and October 2019. At that time, all the patients had at least 3 years of follow-up (Maximum 10 years). Participants were required to assess the change between the first CGA visit and their last follow-up interview on several clinical outcomes using a 5-point Likert scale (1 = much improved, 2 = improved, 3 = no change, 4 = worsened, 5 = much worse). The parameters for evaluation were overall satisfaction from the PDP approach, functional, mental, and cognitive status, nighttime sleep quality, daytime sleepiness, appetite, pain, and incontinence. These were all *subjective* scores, based on the patient’s perception, or family/primary caregiver’s impression when patients had severe dementia or disability. Patients/families were also asked to report *objective* parameters: all types of medications taken, new diagnoses, complications, and hospitalizations since the first CGA visit. These were confirmed by medical documents. Mortality was assessed based on formal data obtained from the Ministry of Internal Affairs through October 2019. The study was started in 2009 and ended in October 2019.

The study protocol was approved in 2009 by the ethics committees of the Shoham Geriatric Medical Center, Pardes-Hana, and later the Wolfson Medical Center, Holon, Israel (ID 0068-15-WOMC SERIAL No. 57077, 9/7/2015). Being an improved quality CGA stressing on the evaluation of medications, a written informed consent was not required by the ethics committees. Only the authors (researchers) were aware of the specific demographic and medical details, and confidentiality of all patients was maintained throughout the study.

### Statistical analysis

Data were analyzed with IBM SPSS statistics software version 25.0 (SPSS Inc., Chicago, IL, USA). All tests were two-tailed and the significance levels were set at 0.05. Baseline characteristics and chronic diseases were presented as means and standard deviations for continuous variables and as frequencies and percentages for categorical variables. Spearman correlations were calculated for continuous variables.

The study population was divided into two groups based on the rate of de-prescribing. The poly-de-prescribers group (PDP) included those who discontinued more than 50% of the drugs taken at the baseline visit, and the control group was termed the non-responders (NR) group, which included older people who discontinued 50% or less of the medications they were taking at the baseline visit.

The chi-square tests were performed to compare the changes in the main clinical outcomes and QoL parameters between groups (PDP group Vs. NR group). Independent t-tests were performed to compare the two groups for continuous variables. For our observational study, the assignment of subjects into groups was not random but rather done based on the number of medications de-prescribed. In an attempt to reduce the group assignment bias and mimic randomization in order to create groups that are comparable on all observed covariates, we adjusted for the propensity score (PPS). We calculated PPS using a logistic regression model with the observed confounders at baseline (age, gender, family status, number of children, health and functional status at baseline, and number of diseases and prescribed drugs at baseline). Odds ratio (OR) and 95% confidence intervals for PDP were compared to NR for the main outcomes (patient/family satisfaction and clinical outcomes, as dichotomous variables improved/not improved) and were adjusted for PPS. The length of follow-up was calculated as the time from the first baseline visit until death or until the last follow-up visit, in those who were still alive. We used the Cox proportional hazards regression models to evaluate the hazard ratios (HR) and 95% confidence intervals for death among groups, adjusted to PPS. The only variable entered into the model was PPS (which already includes different variables as explained in the Methods section).

## Results

A total of 307 patients met the inclusion criteria for the study. The mean age of the patients was 83 years (SD = 5.95; range 65–99 years), and 35% were men. The extent of multi-morbidity on baseline visit was reflected by a mean number of diseases/geriatric syndromes of 10.4 (SD = 2.9, range 2–18); the mean number of drugs at baseline was 9.8 (SD = 2.6, range 6–20), thus reflecting the extent of polypharmacy. The Mini Mental State Examination (MMSE) score was 23.3 ± 8.5 (range 5–30); the Geriatric Depression Scale (GDS) score was 6.94 ± 4.35 (range 1–14). There was no significant difference between men and women for these characteristics. Data on weight were available in 215 participants (range 39–110 kg); it was significantly higher in men as compared to women (77 ± 12 versus 64 ± 12 kg, respectively).

The study population was consuming a variety of drugs from several groups, often more than one medication for the same indication (particularly antihypertensive medications [AHT]). Table 1 describes the most common medications used by all participants at baseline visit, the number of those de-prescribed, and the number of medications that have actually been discontinued at the end of follow-up. For instance, out of 243 participants who were on statins, 154 (63%) eventually stopped using them. Stopping slow-release nitrates was recommended in 19 patients out of 20 who were taking them and achieved in 13 (68%); none of them experienced angina pectoris or electroencephalogram (ECG) changes, and no one needed the sublingual nitroglycerin that had been provided as a means of precaution for PRN (*pro re nata*, as needed) use. None of the older patients for whom PPIs or H2 blockers were de-prescribed experienced gastrointestinal bleeding. Out of 73 patients who were diagnosed as having Parkinson’s disease, none of the 23 (32%) patients in whom anti-Parkinsonian medications had been de-prescribed experienced deterioration in function or in extrapyramidal symptoms or signs. Subsequent reductions in serum hemoglobin concentration were not found in any of those in whom iron was stopped.

**Table 1:** Rate of de-prescribing for specific medications/drug groups.^†^

	Total *n* = 307 (%)	De-prescribing suggested (%)	De-prescribing achieved (%)
Calcium channel blockers	136 (44)	127 (93)	63 (46)
Beta-blockers	182 (59)	48 (26)	50 (27)
Angiotensin-converting enzyme inhibitors	112 (36)	81 (72)	51 (45)
Angiotensin II receptor blockers	106 (34)	61 (57)	30 (28)
Hydrochlorothiazide	79 (26)	70 (88)	54 (68)
Furosemide	73 (24)	50 (68)	28 (38)
Spironolactone	22 (7)	15 (68)	10 (45)
Alfa blockers	79 (26)	79 (100)	44 (55)
Clonidine	7 (2)	5 (71)	3 (42)
Slow-release nitrates	20 (6)	19 (95)	13 (65)
Amiodarone	28 (9)	11 (39)	11 (39)
Digoxin	6 (2)	2 (33)	2 (33)
Acetyl Salicylic acid (Aspirin)	186 (61)	133 (71)	104 (55.9)
Warfarin (Coumadin)	46 (15)	13 (28)	16 (34)
Enoxaparin	4 (1)	4 (100)	4 (100)
Clopidogrel	52 (17)	12 (23)	18 (34)
DOAC*	14 (5)	1 (7)	1 (7)
Sulfonylurea	22 (7)	22 (100)	13 (59)
Metformin	75 (24)	43 (57)	23 (30)
Repaglinide	26 (8)	21 (80)	9 (34)
DPP4 inhibitor **	19 (6)	11 (57)	4 (21)
Insulin	26 (8)	3 (11)	2 (7)
Statins	243 (79)	235 (96)	154 (63)
Fibrates/Ezetimibe	14 (5)	14 (100)	4 (28)
Thyroid hormones	72 (23)	1 (1.4)	7 (9.7)
Allopurinol	17 (5)	11 (64)	9 (53)
Proton pump inhibitors (PPI)	173 (56)	151 (87)	73 (42)
H2 Blockers	37 (12)	35 (94)	24 (64)
Benzodiazepines	219 (71)	205 (93)	126 (57)
Z-Drugs ***	43 (14)	24 (55)	17 (39)
SSRIs/SNRIs ^^	124 (40)	118 (95)	65 (52)
Trazodone	6 (2)	3 (50)	4 (66)
Amitriptyline	9 (3)	7 (77)	0 (0)
Mirtazapine	38 (12)	18 (47)	15 (39)
Duloxetine	24 (8)	10 (41)	8 (33)
Diphenyl-hydantoin (Phenytoin)	1	1 (100)	1 (100)
Carbamazepine	3 (1)	2 (66)	2 (66)
Valproate	5 (2)	1 (20)	2 (40)
“Other” Anti-Epileptics ^^^	9 (3)	6 (66)	4 (44)
Haloperidol	3 (1)	1 (33)	1 (33)
Anti-Psychotics (typical and atypical)	38 (12)	24 (63)	17 (44)
Anti-Vertigo Drugs ^	22 (7)	21 (95)	16 (72)
Amantadine	11 (50)	8 (72)	4 (36)
Levodopa-Carbidopa	41 (13)	19 (46)	10 (24)
Other anti-Parkinson’s #	20 (6)	11 (55)	9 (45)
medicines for dementia ##	72 (23)	65 (90)	43 (59)
NSAIDs @	26 (8)	24 (92)	14 (53)
Paracetamol	10 (3)	1 (10)	1 (10)
Dipyrone	26 (8)	5 (19)	3 (11)
Tramadol	15 (5)	8 (53)	7 (46)
Pregabaline	11 (4)	6 (54)	7 (63)
Opioids	18 (6)	7^†^ (38)	6 (33)
Oxybutynin	18 (6)	14 (77)	10 (55)
Trospium chloride	21 (7)	10 (47)	11 (52)
Dutasteride	29 (9)	6 (20)	9 (31)
Laxatives	112 (36)	3 (2)	20 (17)
Pentoxifylline	2	2 (100)	2 (100)
Steroids (Oral)	14 (5)	6 (42)	5 (35)
Steroid Inhalers	49 (16)	6 (12)	11 (22)
Bisphosphonates	71 (23)	37 (52)	25 (35)
Melatonin controlled release (Circadin)	12 (4)	1 (8)	3 (25)
Anti-allergic	23 (7)	2 (8)	4 (17)
Iron Preparations	48 (16)	32 (67)	15 (31)
Over the counter (OTC) compounds^†^			
Calcium supplements	132 (43)	23 (17)	42 (32)
Vitamin D	193 (63)	14 (7.2)	49 (25)
Vitamin B12	50 (16)	7 (14)	11 (22)
Folic Acid	47 (15)	11 (23)	19 (40)
Multi-vitamins	131 (43)	27 (21)	22 (17)

^†^The last five over the counter (OTC) vitamins and minerals were NOT included in the total drug count. *DOAC, Direct Oral Anticoagulants (Dabigatran, Apixaban, Rivaroxaban), **DPP4INH, Dipeptidyl peptidase-4 inhibitor (Sitagliptin, Vildagliptin), ***Z-drugs, non-benzodiazepine sleep inducers (Zolpidem, Zopiclone). ^Anti Vertigo drugs = Betahistine Dihydrochloride, Cinnarizine, Sulpiride. ^^SSRI, Selective Serotonin Reuptake Inhibitor; SNRI, Serotonin–Norepinephrine Reuptake Inhibitors. ^^^Other Anti-Epileptics = Levetiracetam, Lamotrigine, Primidone, Topiramate. ^#^Other Anti-Parkinson’s = Amantadine, Selegiline, Rasagiline Pramipexole. ^##^Anti-Alzheimer’s = to “improve memory” (donepezil, memantine, galantamine, rivastigmine). ^@^NSAIDs, Nonsteroidal anti-inflammatory drugs (Ibuprofen, diclofenac, indomethacin).

In the subgroup analysis of objective parameters, 146 patients reduced the number of drugs by more than 50% from baseline (designated the PDP group) and 161 patients reduced the number of drugs by 50% or less (NR, control group). The PDP group was significantly older than the NR group (84.45 ± 5.76 vs. 81.85 ± 5.86; *p* < 0.001). The PDP group had lower rate of hypertension and higher rate of incontinence and dementia (Table 2). With regard to all other health problems, the groups were comparable.

**Table 2:** Prevalence of chronic diseases, geriatric syndromes/symptoms in the study (PDP) and control (NR) groups^*^

Chronic disease/Syndrome	Total, *n* = 307	Non-responders NR, *N* = 161	Poly-de-prescribers PDP, *N* = 146	*p*
Hypothyroidism	69 (22.5)	42 (26)	27 (18.5)	0.111
Diabetes mellitus	119 (38.8)	70 (43.5)	49 (34)	0.075
Hyperlipidemia	236 (76.9)	127 (79)	109 (75)	0.381
Hypertension	246 (80.1)	136 (84.5)	110 (75)	**0.045**
Ischemic heart disease	94 (30.6)	50 (31)	44 (30)	0.862
Congestive heart failure	22 (7.2)	8 (5.0)	14 (10.0)	0.117
Atrial fibrillation	73 (23.8)	37 (23)	36 (25)	0.730
Peripheral vascular disease	23 (7.5)	12 (7.5)	11 (7.5)	0.979
Cerebral stroke	79 (26.0)	41 (25.5)	38 (26)	0.911
Chronic obstructive lung disease	37 (12.1)	22 (14)	15 (10)	0.362
Chronic renal failure	44 (14.3)	21 (13)	23 (16)	0.499
Benign prostatic hypertrophy	68 (22)	37 (23)	31 (21)	0.713
Urine incontinence	138 (45.0)	63 (39)	75 (51)	**0.031**
History of malignancy	62 (20.2)	29 (18)	33 (23)	0.317
Osteoporosis	135 (44)	68 (42)	67 (46)	0.519
Recurrent falls	193 (62.9)	96 (60)	97 (66)	0.217
Parkinson’s disease (Parkinsonism)	41 (13.4)	21 (13)	20 (14)	0.866
Sleep disorders	233 (75.9)	123 (76)	110 (75)	0.826
Depression	149 (48.5)	71 (44)	78 (53)	0.103
Anxiety	91 (29.6)	48 (30)	43 (29.5)	0.945
Dementia	69 (22.5)	21 (13)	48 (33)	**<0.001**

^*^As written in the medical file, in brackets percentage (%) of group. Bold values are used to highlight statistical significance.

Table 3 summarizes the objective long-term effects of poly-de-prescribing. Both groups were followed up for an average of 57 months.

**Table 3:** Long-term objective end points of PDP (number of drugs, hospitalizations, and survival).

	Total *N* = 307	NR* *N* = 161	PDP* *N* = 146	*p*-value
Follow-up (months)	57 ± 30	58 ± 30	55 ± 29	0.319
No. of drugs at baseline	9.8 ± 2.6	10 ± 2.6	9.5 ± 2.5	0.021
No. of drugs suggested to de-prescribe	6.2 ± 2.1	6 ± 1.9	6.5 ± 2.3	0.021
No. of drugs for which de-prescribing was achieved	4.4 ± 3.1	2.2 ± 2.1	6.7 ± 2.0	<0.001
Rate of de-prescribing**	46% ± 30%	22% ± 19%	72% ± 14%	<0.001
Hospitalizations (*n* = 282)	117 (48.5%)	54 (44%)	63 (53%)	0.141
Deaths	188 (61%)	100 (62%)	88 (60%)	0.741
Survival rate after 3 years of follow-up (months)	73% ± 3.7%	73 ± 3.5%	73% ± 3.7%	0.940

*NR, Non-Responders; PDP, Poly de-prescribing study group. **Rate of de-prescribing = No. of Medications stopped at last visit/No. of Medications at baseline.

**Figure 2:**
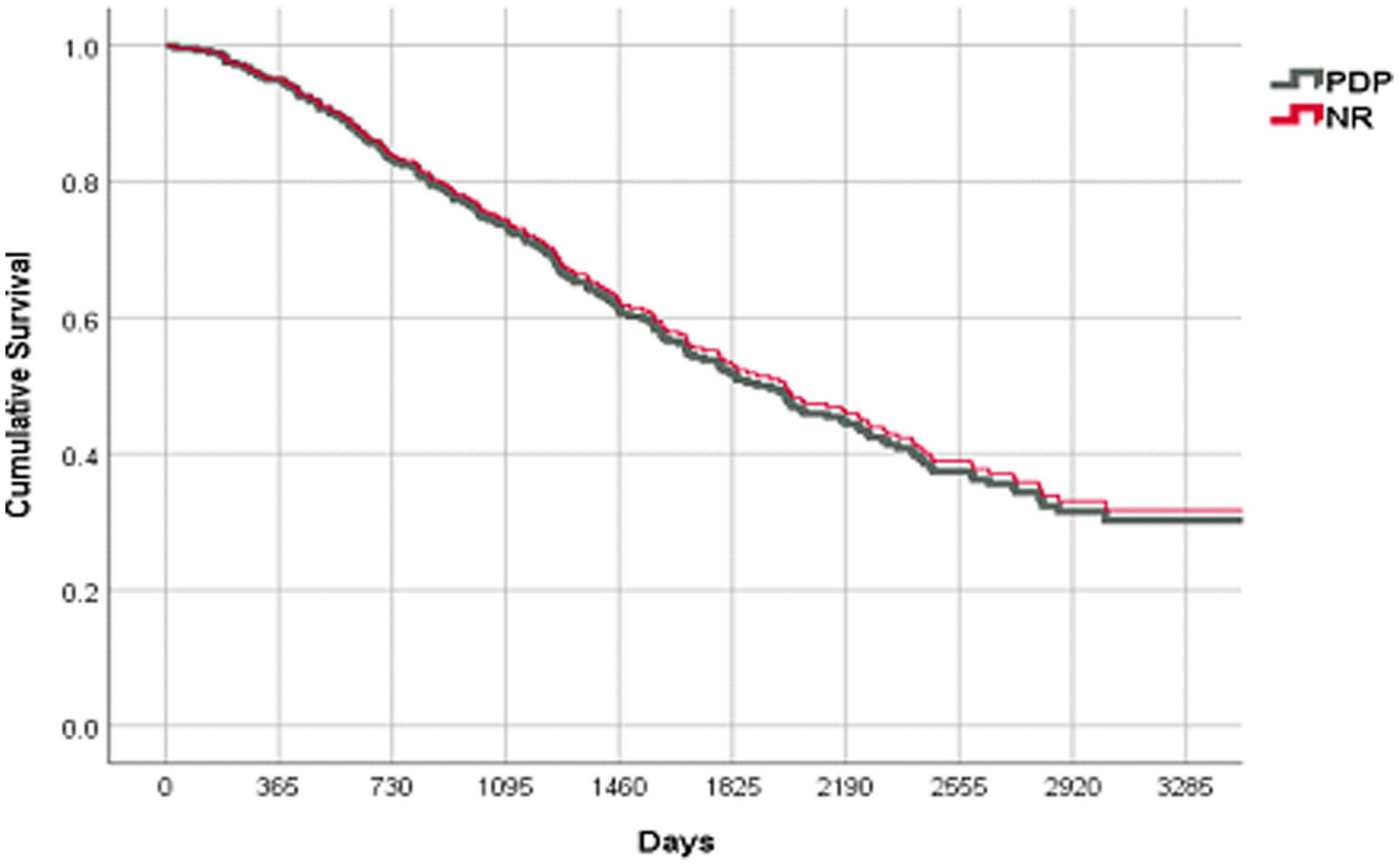
Survival curves: PDP vs. control groups.

In spite of a significantly higher number of de-prescribed medications in the PDP group, there was no change in the objective end points and no increase in the rate of hospitalizations or mortality. The PPS-adjusted Cox proportional survival curves were the same for both groups (Figure 2, OR 0.960; 95% C.I. [0.695–1.324], *p* = 0.802).

Out of the 307 participants enrolled in the study at the first visit, completely reliable full end-of-study questionnaires could not be obtained from 25 participants. Therefore, we performed the *objective* parameter analysis for all the 307 participants of the cohort study (Tables 1–3), but comparison of the *subjective* parameters was based on data from 282 participants only (Table 4).

**Table 4:** Long-term subjective end points of Poly-de-prescribing (adjusted to PPS)^†^.

A. Comparison of specific clinical outcomes between groups						
	Total *N* = 282 (%)	NR *N* = 142 (%)	PDP* N = 140 (%)	NR*	PDP* OR (95% C.I.)	*p*-value
High patient satisfaction with medical change	182 (59)	57 (35)	125 (86)	1.0	9.5 (4.95–18.4)	<0.001
Health status improved	103 (34)	41 (26)	62 (42)	1.0	2.19 (1.27–3.77)	0.001
Functional status improved	51 (18)	16 (11)	35 (25)	1.0	2.43 (1.21–4.87)	0.012
Cognitive status improved	22 (7.5)	9 (6.3)	13 (9)	1.0	3.32 (1.93–5.71)	<0.001
Mental status improved	116 (41)	39 (27.5)	77 (55)	1.0	1.23 (0.47–3.19)	0.672
Nighttime sleep quality improved	86 (30.5)	25 (17.5)	61 (43)	1.0	3.38 (1.88–6.09)	<0.001
Daytime wakefulness improved	50 (18)	13 (9)	37 (26)	1.0	2.98 (1.44–6.15)	0.003
Urine continence improved	10 (3)	2 (1.4)	8 (5.7)	1.0	1.46 (0.54–3.96)	0.461
Appetite improved	58 (21)	23 (16)	35 (25)	1.0	5.43 (1.02–29.03)	0.048
Pain decreased	20 (7)	9 (6)	11 (8)	1.0	1.41 (0.79–2.80)	0.221

B. Comparison of initiation, length, and extent of clinical improvement**						
	Total *N* = 282 (%)	NR* N = 142 (%)	PDP* N = 140 (%)	NR	PDP OR (95% C.I.)	*p*-value
Improvement within 3 months	148 (52.5)	49 (34.5)	99 (71)	1.0	4.33 (2.51–7.46)	<0.001
Improvement persisted for more than 1 Year	146 (52)	49 (35)	97 (70)	1.0	3.92 (2.29–6.73)	<0.001
No worsening after 2 Years	166 (60)	67 (48)	99 (72)	1.0	2.36 (1.37–4.06)	0.002
Family doctor response accepted completely^***^	155 (55)	43 (30)	112 (80)	1.0	7.54 (4.24–13.39)	<0.001

^†^Full questionnaires could not be obtained from 25 participants. Therefore, the findings in this table are based on data from 282 participants only. ^*^NR, Non-Responders; PDP, Poly de-prescribing study group. ^**^Improvement = Improved + much improved on the 5-point Likert scale (see Methods, follow up). ^***^The Family Doctor accepted all recommendations for de-prescribing.

We observed more significant improvements in outcomes among the PDP group as compared to the NR group in terms of patient/family satisfaction, self-perceived health, functional and cognitive status as well as nighttime sleep quality and daytime wakefulness. No subjective deterioration was observed for any of the outcomes in the PDP group as compared to the NR group (Table 4). Although the rate of mental status improvement was higher among the PDP group patients in univariate analysis (two times more improvement), the difference between the groups was not statistically significant in the multivariate analysis (possibly due to relationships to other variables in the model). As for the initiation, length, and extent of clinical improvement, high satisfaction and significant improvement in clinical outcomes were already apparent in 71% of the patients in the PDP group within the first 3 months of follow-up as compared to only 34.5% of the patients in the NR group; the improvement persisted more than one year in 70% of the PDP participants as compared to only 35% in the NR group (*p* < 0.001 in both parameters). Among those patients surviving longer than 2 years, 52% in the NR group reported a worsening in their clinical status compared to only 28% of the PDP group (*p* < 0.001).

## Discussion

Modern medicine has prolonged life expectancy and with it a growing VOCODFLEX population (as defined by us previously) represented by very old people, with comorbidity, dementia, frailty/disability, and/or limited life expectancy. This population poses new challenges to the medical community such as the worldwide problem of inappropriate medication use and polypharmacy (IMUP). Most previous attempts to combat IMUP were unsuccessful in reducing its negative medical, nursing, and socioeconomic effects (3, 10, 11, 21, 22). This may be in part due to the fact that strategies employed to fight IMUP were based on a single-disease–single-drug model, which assumes that patients are largely homogeneous. This is incongruent with the reality of older populations where heterogeneity is the norm and where there is no longer a “natural clinical course of disease,” owing to the inseparable co-mingling of multiple diseases with multiple drugs (1, 3, 21). Older people, particularly the VOCODFLEX group, deserve a different clinical approach as they present unique challenges.

The etiology of IMUP is multifactorial. Older people are excluded from randomized controlled trials (RCTs) and the few trials in older people are non-representative of the general old population (23, 24). Therefore, applying all guidelines in older people may not necessarily lead to an improvement in the quality of care, and sometimes even cause greater harm than good (25). It may fuel vicious cycles of “prescription cascades” where symptoms resulting from IMUP are perceived as “new diseases,” leading to futile evaluations and over-diagnosis (26), thus making the spread of IMUP inevitable.

There are many tools to assess the appropriateness of prescribing (27). However, even the most sophisticated computer-assisted methods (10) as well as lists of “drugs to avoid” (e.g., Beers criteria, START/STOPP) have failed to show significant improvements in clinical outcomes, rendering them insufficient as a standalone approach against IMUP (2, 3, 11). In our view, all these “drugs to avoid” approaches are basically flawed. Apart from an unbearable rate of false-positive alerts, separating “bad drugs” from “good drugs” may be dangerous, providing false reassurance to clinicians and concealing the damage caused by the interactions between the remaining, apparently “appropriate” drugs (19, 22).

In this study, we show that even “appropriate drugs” may become harmful when they accumulate in the system; stopping such “appropriate drugs” may contribute to the overall improvement achieved by PDP.

Gnjidic et al. (13) and Rausch et al. (14) have suggested that the number of different medications, starting from three or five, is associated with an increased likelihood of serious adverse drug effects (ADE). If the sum of all negative outcomes of polypharmacy (sometimes unrecognized) outweighs the potential benefits gained from every specific drug, then PDP can be the first step toward implementing the dictum “first, do not harm.”

Our study represents the first longitudinal observational study in older people with polypharmacy, evaluating the effect of de-prescribing on long-term clinical outcomes and QoL.

The Garfinkel method we employed for de-prescribing has already been implemented in nursing departments (20) and in community-dwelling elders (1, 19) and exhibited safety and efficacy while achieving sustained improvements in clinical outcomes in both. In this research, participants who best complied with de-prescribing and stopped more than 50% of their medications represented the study group (PDP). The non-responders (NR) group included patients who continued taking medications as before or more of them, or discontinued less than 50% of their initial drugs. Unlike our previous research where we used a cutoff of three medications to compare two groups, in this study, we chose to look at the percentage of drugs de-prescribed rather than the absolute number of medications. This may seem confusing because by achieving significant de-prescribing of nine drugs, a patient may still be classified among the non-responder, control group if the number of drugs taken in the first place was more than 18. We chose 50% as a cutoff between the two groups in order to check whether we really need to reduce a substantial amount of medications in order to observe any beneficial effect. We found that regardless of the number of drugs consumed, de-prescribing as many medications as possible (PDP) is beneficial.

When isolating all other factors, we showed in this study that the family doctor’s willingness to follow through with de-prescribing recommendations was the most influential factor (*p* < 0.001) on the decision of the patient/family if and to what extent to adopt de-prescribing. The GP actually determined the patient’s group (PDP or NR) and the consequences of this choice on the patient’s clinical outcomes (Table 4).

The finding that in most cases where PDP is implemented improvement appears quickly—within 3 months following PDP with no worsening even after 2 years of follow-up—is encouraging. Combined with the prompt positive impact, the long-term benefits, such as sustained improvement over several years, highlight the enduring positive effects of PDP. Many times, patients/families themselves wish to broaden the spectrum of PDP, which highlights yet another beneficial medico-legal advantage of this method. All these enablers should help overcome common barriers that underlie patient’s and doctor’s fear of routine de-prescribing (12, 28).

The goal of stopping as many drugs as possible simultaneously does not include medications that are “life-saving” or improve QoL in specific subpopulations to which the patient belongs. As life expectancy decreases particularly in the VOCODFLEX population, the role of preventative drugs and the positive benefit/risk ratio of most medications is declining (29–31). Indeed, in our study, mortality rate was not increased even after years following PDP.

Most physicians are reluctant to de-prescribe “simultaneously” and prefer stopping drugs one by one. This reaction is rooted in our traditional “single-drug model” perception. However, the “one drug at a time” approach is inappropriate for the practice of de-prescribing. Facing “multi-disease multi-drug” situations, the risk of IMUP is increasing in correlation to the number of drugs. It is the combination of many drugs that result in the negative effects of IMUP, and this study shows that removing the largest possible combinations bestows the greatest benefit. Furthermore, as Holmes and others state, we may not have time to stop drugs one by one facing the unknown but limited life expectancy of these patients (30, 31).

In our perception, it is not important to know what drug combinations that were removed caused an improvement in each patient. The important issue is that we have successfully achieved the main goals of medicine: symptomatic improvement, better QoL, and patient/family satisfaction.

## Strengths and limitations

This study is the first longitudinal research evaluating the long-term beneficial effects of poly-de-prescribing in terms of the extent of clinical benefits and improvement in the quality of life of older people in polypharmacy.

An important limitation of this study is the lack of randomization and a true “traditional” control group. The cohort represents a group of people who were already dissatisfied by their current health situation and treatment and who chose to consult the geriatrician for a second opinion. This cohort therefore represents a self-selected target group, which may have influenced the impact of the PDP. All participants received recommendations based on the same algorithm, but compliance varied among them. A “pure” RCT would require de-prescribing many drugs and comparing outcomes in patients who did not have the same drugs removed. Considering the complexity of old patients’ characteristics and limited life expectancy, performing a true RCT would be unrealistic. However, this preliminary “proof of concept” observational cohort study may serve as a basis for planning future randomized PDP studies.

Our “subjective results” (Tables 3, 4) are based on participants’ opinion of how general health and specific conditions may have changed. The study could benefit from objective measures for clinical outcomes. Rather than using patient/family opinion for measuring subjective parameters, it would probably be better to use instruments that assess the overall quality of life (or components thereof) at both time points, not only at baseline before the intervention but also at a specified later time point (last follow-up). On the other hand, the fact that objective parameters showed no significant differences between the groups, while subjective parameters exhibited notable improvements in the PDP group, suggests that patient-reported outcomes and satisfaction play a crucial role in assessing the effectiveness of PDP. The lack of statistical significance in mental status improvement between groups in multivariate analysis warrants further investigation using larger samples.

Another limitation is that adverse symptoms such as falls have not been evaluated as outcomes and compared between the two groups.

One may argue that study participants might have had social desirability bias to report improved outcomes in the survey questionnaires. However, as all the participants responded to the same questionnaire, we do not believe this could result in a major bias in the study. Another limitation to consider is that all the participants consulted the same geriatrician, making it difficult to distinguish between the benefit of the algorithm and the impact of the geriatrician’s skill. This aspect could be of interest to others who may wish to replicate this work. Therefore, one should be cautious in deducing our results to the entire elderly population. The assertion that the clinical harm outweighs all beneficial effects without distinguishing between different types of medications and their potential individual impacts may be overly generalized. It is essential to recognize that not all drugs contribute equally to harm, and certain medications may exert distinct influences on clinical outcomes. The consideration of factors such as drug interactions, patient adherence, and individual characteristics may provide a better understanding.

For many older people including VOCODFLEX, poly-de-prescribing is a key clinical priority to prevent further morbidity/mortality. Routine drug re-evaluation is an essential part of CGA (32); the Garfinkel Algorithm should therefore be perceived not as a new intervention but rather an improved “medication debridement” tool, that should be used in a rational, guided, yet aggressive way (19, 20). This algorithm also adopts the 2012 recommendations of the Institute of Medicine (33): “Focus on QoL outcome measures, take a more coordinated approach to meeting both health and social needs”, highlighting the shift in emphasis to ‘living well’ rather than reducing mortality (34). PDP wouldn’t be necessary if periodic medication reviews were performed and medications stopped when necessary. Furthermore, we should change our “all drugs forever” attitude and educate all health professionals (35) as well as the general population (36, 37) stressing that every prescription should be viewed as a time-limited intervention. In line with many studies showing the negative health outcomes of IMUP, Fabbietti et al. (38) have proven that “Hyperpolypharmacy” is associated with functional decline. At the moment we can’t offer supporting evidence or rationale for these findings but in the future, in order to enhance this hypothesis it would be crucial to elucidate the mechanisms through which the absolute number of drugs contributes to IMUP. Nevertheless, it may be concluded that deprescribing in itself is usually associated with a significant clinical economical win-win situation (39).

## Data availability statement

The original contributions presented in the study are included in the article/Supplementary material, and further inquiries can be directed to the corresponding author.

## Ethics statement

The studies involving humans were approved by ethics committees: Shoham Medical Geriatric Center, Pardes Hana, Israel & Wolfson Medical Center, Holon, Israel. The studies were conducted in accordance with the local legislation and institutional requirements. The ethics committee/institutional review board waived the requirement of written informed consent for participation from the participants or the participants’ legal guardians/next of kin because both ethics committees determined that an unwritten consent was sufficient (approval given by all participants or legal guardians).

## Author contributions

DG: Conceptualization, Data curation, Formal analysis, Investigation, Methodology, Project administration, Software, Supervision, Validation, Writing – original draft, Writing – review & editing. YL: Data curation, Formal analysis, Investigation, Software, Supervision, Validation, Writing – review & editing.
