# Supplementary material for: Optimizing clinical outcomes in polypharmacy through poly-de-prescribing: a longitudinal study
Source: Front Med (Lausanne). 2024 Apr 30;11:1365751. doi: 10.3389/fmed.2024.1365751 (PMC11091405; doi:10.3389/fmed.2024.1365751)
Supplement: Supplementary file 1 [file Data_Sheet_1.docx]

**Appendix 1**

**Subjective Evaluation of the GPGP Efficacy and Safety**

**Questionnaire**

# (to be filled at the Last Follow up visit)

**Patient ID No**. : ___ ___ ___ ___ ___ ___ ___ ___ ___

**Date filled** : _ _/_ _/_ _

1. Person filling the questionnaire
   - ( ) patient
   - ( ) spouse
   - ( ) son/daughter
   - ( ) other _____________________

1. What was the patient's Health Status in the first Baseline visit ? •
2. * ( ) excellent
   - ( ) good
   - ( ) fair
   - ( ) Bad
   - ( ) very bad

1. What is the patient's Health Status today (Last follow up)?
   - ( ) excellent
   - ( ) good
   - ( ) fair
   - ( ) Bad
   - ( ) very bad

What is the Level of your Satisfaction from the De-Prescribing Intervention ?

- - ( ) very high
  - ( ) high
  - ( ) medium
  - ( ) low
  - ( ) very low

1. What was the patient's Functional Status in the first Baseline visit ?
   - ( ) independent
   - ( ) frail
   - ( ) mild disability (needs help in maximum 2 ADL *, no incontinence)
   - ( ) disability (needs help in ≥3 ADL *, urine or double incontinence)
   - ( ) Severe Disability/Bed ridden

* ADL = Activity of daily living.

1. What is the patient's Functional Status today (Last Follow up) ?
   - ( ) independent
   - ( ) frail
   - ( ) mild disability (needs help in maximum 2 ADL *, no incontinence)
   - ( ) disability (needs help in ≥3 ADL *, urine or double incontinence)
   - ( ) Severe Disability/Bed ridden

* ADL = Activity of daily living.

1. Following Drug intervention, is there any Change in Functional Status ?
   - ( ) significant improvement
   - ( ) improvement
   - ( ) No change
   - ( ) worse
   - ( ) significantly worse

1. Following Drug intervention, is there any Change in Mental Status ?

(Mood, Depression)

- - ( ) significant improvement
  - ( ) improvement
  - ( ) No change
  - ( ) worse
  - ( ) significantly worse
  - ( ) unknown / not relevant

1. Following Drug intervention, is there any Change in Cognitive Status ?

(memory, orientation, communication, judgment)

- - ( ) significant improvement
  - ( ) improvement
  - ( ) No change
  - ( ) worse
  - ( ) significantly worse
  - ( ) unknown / not relevant

1. Following Drug intervention, is there any Change in Night Sleep Quality ?
   - ( ) significant improvement
   - ( ) improvement
   - ( ) No change
   - ( ) worse
   - ( ) significantly worse
   - ( ) unknown / not relevant

1. Following Drug intervention, is there any Change in Daily Sleepiness ?
   - ( ) significant improvement
   - ( ) improvement
   - ( ) No change
   - ( ) worse
   - ( ) significantly worse
   - ( ) unknown / not relevant

1. Following Drug intervention, is there any Change in Appetite ?
   - ( ) significant improvement
   - ( ) improvement
   - ( ) No change
   - ( ) worse
   - ( ) significantly worse
   - ( ) unknown / not relevant

1. Following Drug intervention, is there any Change in Urine Continence ?
   - ( ) significant improvement
   - ( ) improvement
   - ( ) No change
   - ( ) worse
   - ( ) significantly worse
   - ( ) unknown / not relevant

1. What was the Family Doctor's Reaction to the De-prescribing Plan ?
   - ( ) accepted the plan completely
   - ( ) accepted mostly
   - ( ) accepted partially
   - ( ) refused most recommendations to De-prescribe medications
   - ( ) refused all recommendations to De-prescribe medications
   - ( ) the doctor refused but the patient/family decided to accept recommendations

1. Following Initiation of the De-prescribing Plan, when did the improvement begin ?
   - ( ) within a month
   - ( ) 2-3 months following Initiation of De-prescribing
   - ( ) more than 3 months later
   - ( ) there was no improvement at all

1. If Improvement Occurred, How Long has it Persisted ?
   - ( ) more than 2 years
   - ( ) 1-2 years
   - ( ) 6-12 months
   - ( ) 3-6 months
   - ( ) less than 3 months

1. If Worsening was noticed following De-prescribing, When did it begin ?
   - ( ) No worsening
   - ( ) after more than 2 years
   - ( ) after 1-2 years
   - ( ) after 6-12 months
   - ( ) after 3-6 months

•

1. Has the patient been Hospitalised since the first Baseline visit?
   - ( ) No
   - ( ) Yes
   - ( ) How many times # _ _

# please send documents from the hospital

1. Do you want to add any Comment ?

Please send an Updated list of All the Patient's Medications (to e-mail ….. or Fax …..)

End of questionnaire

*********************
